# Vitamin D deficiency and psychotic features in mentally ill adolescents: A cross-sectional study

**DOI:** 10.1186/1471-244X-12-38

**Published:** 2012-05-09

**Authors:** Barbara L Gracious, Teresa L Finucane, Meriel Friedman-Campbell, Susan Messing, Melissa N Parkhurst

**Affiliations:** 1 Center for Innovation in Pediatric Practice, The Research Institute at Nationwide Children’s Hospital and The Ohio State University, Columbus, OH, USA; 2 Clinical Research Coordinator, University of Rochester Medical Center, Department of Psychiatry, 300 Crittenden Boulevard, Rochester, New York, 14642, USA; 3 Psychiatric Nurse Practitioner, Irwin Army Community Hospital Behavioral Health, 600 Caisson Hill Road, Fort Riley, KS, 66442, USA; 4 Nationwide Children’s Hospital 700 Children’s Drive, Columbus, OH, 43205, USA; 5 Senior Research Associate, Department of Biostatistics and Computational Biology, University of Rochester Medical Center

**Keywords:** Vitamin D, Adolescents, Deficiency, Psychosis

## Abstract

**Background:**

Vitamin D deficiency is a re-emerging epidemic, especially in minority populations. Vitamin D is crucial not only for bone health but for proper brain development and functioning. Low levels of vitamin D are associated with depression, seasonal affective disorder, and schizophrenia in adults, but little is known about vitamin D and mental health in the pediatric population.

**Methods:**

One hundred four adolescents presenting for acute mental health treatment over a 16-month period were assessed for vitamin D status and the relationship of 25-OH vitamin D levels to severity of illness, defined by presence of psychotic features.

**Results:**

Vitamin D deficiency (25-OH D levels <20 ng/ml) was present in 34%; vitamin D insufficiency (25-OH D levels 20–30 ng/ml) was present in 38%, with a remaining 28% in the normal range. Adolescents with psychotic features had lower vitamin D levels (20.4 ng/ml vs. 24.7 ng/ml; p = 0.04, 1 df). The association for vitamin D deficiency and psychotic features was substantial (OR 3.5; 95% CI 1.4-8.9; p <0.009). Race was independently associated with vitamin D deficiency and independently associated with psychosis for those who were Asian or biracial vs. white (OR = 3.8; 95% CI 1.1‒13.4; p < 0.04). Race was no longer associated with psychosis when the results were adjusted for vitamin D level.

**Conclusions:**

Vitamin D deficiency and insufficiency are both highly prevalent in adolescents with severe mental illness. The preliminary associations between vitamin D deficiency and presence of psychotic features warrant further investigation as to whether vitamin D deficiency is a mediator of illness severity, result of illness severity, or both. Higher prevalence of vitamin D deficiency but no greater risk of psychosis in African Americans, if confirmed, may have special implications for health disparity and treatment outcome research.

## Background

Vitamin D deficiency is endemic across the life span and in diverse populations throughout the world [1]. Contributing factors are lack of exposure to sunlight and insufficient dietary intake; individuals with darker skin are at higher risk due to low cutaneous synthesis and dairy-poor diets. In a study of healthy Northeastern US adolescents, more than 90% of African American teens and 55% of all teens had low vitamin D [2]. The National Health and Nutritional Examination Survey (NHANES 2001–2004) found an overall US prevalence of vitamin D insufficiency in adolescents of 61%, with 9% deficient [3].

Vitamin D is well recognized as essential for intestinal calcium absorption, serum calcium homeostasis, optimal skeletal development, and the prevention of rickets and osteoporosis [4]. The importance of vitamin D to the CNS in both healthy and psychiatric populations is less well-appreciated and is vastly understudied compared to its known impact on bone health. Vitamin D receptors are present throughout the brain, and D-deficiency is associated with negative CNS effects in animal studies [5]. Vitamin D receptors and activating enzymes are particularly prominent in the hypothalamus and substantia nigra, and are involved in glucocorticoid signaling in hippocampal cells. Depletion models show maternal offspring with abnormal brain shape, cell number, and reduced neurotropic factors and receptors. Vitamin D receptor animal knock-out models show increased anxiety, decreased activity, and muscular and motor impairments, resembling phenotypic models of depression. Vitamin D is neuroprotective to hippocampal cells, through regulating calcium ion channels and activating PKC and mapPK pathways.

Clinical studies reinforce the significance of this basic work. A Finnish cohort supplemented with prenatal and infant vitamin D demonstrated reduced adult risk for schizophrenia [6]. Low vitamin D levels were found to correlate with major depression [7] and premenstrual mood symptoms in women [8], and mood disorders and cognitive impairment in older adults [9]. Two randomized controlled trials (RCT) have shown that raising vitamin D levels improved depression. The first study examined phototherapy vs. vitamin D supplementation for seasonal affective disorder, and found a positive effect for vitamin D via either supplementation or phototherapy within one month [10]. Another RCT of overweight and obese subjects, at greater risk for low vitamin D than those of normal weight, found higher levels of depression with low vitamin D; supplementation resulted in significant improvement in depressive symptoms after one year [11]. To the best of our knowledge, there have been no published studies examining vitamin D deficiency and the presence of psychosis in adolescents.

We hypothesized that, in severely mentally ill adolescents, defined as adolescents requiring either inpatient or partial hospitalization, 1) rates of vitamin D insufficiency and deficiency would be greater than those documented in general US populations, and 2) lower vitamin D levels would be associated with mental illness severity, defined as presence of psychotic features.

## Methods and Materials

### Ethics

The University of Rochester Research Subject Review Board approved the retrospective chart review study.

### Participants

The study population included 75 females and 29 males aged 12 to 18 years admitted to the Strong Behavioral Health Child and Adolescent Acute Inpatient Service or Partial Hospitalization Service (CAPHS), Department of Psychiatry, University of Rochester, NY, between October 2008-February 2010 who had serum 25-OH vitamin D levels collected on routine admission laboratory testing as part of a quality improvement initiative.

### Data collection

Charts were identified by a clinical admission database of the service. Diagnostic evaluations and symptom reports from parents/legal guardians and adolescents were extracted from medical records for the period of clinical care.

### Clinical diagnoses

Clinical DSM-IV diagnoses were predominantly affective disorders (bipolar disorders, N = 37; depressive disorders, N = 36; mood disorder NOS, N = 15; psychotic disorders, N = 8; anxiety disorders, N = 4; and ADHD/ODD N = 1).

### Patient-reported psychosis and potentially related variables

Psychotic symptoms, defined as hallucinations, paranoia, or delusions, were documented on standardized admission assessment forms by the emergency room psychiatrist and the admitting attending. They were categorized dichotomously as yes/no by the second author (TLF), who was blinded to both the purpose of the study and to vitamin D levels until after record extraction was completed. Other variables examined included: race, month of admission/vitamin D level, insurance status, urban/suburban/rural residence, inpatient/partial hospital outpatient, clinical DSM-IV diagnosis, smoking status, age of onset of mental illness, admitting medications, past medications, and immediate and extended family psychiatric and medical history.

### Vitamin D laboratory analysis and categorical definitions

Vitamin D 25-OH (25OHD) levels were analyzed by chemiluminescent immunoassay at ARUP laboratories, SLC, Utah, and recorded as normal if >30 ng/ml, insufficient if 20–30 ng/ml, and deficient if <20 ng/ml, as per expert guidelines [12].

### Statistical analyses

Continuous data were graphically inspected for distributional assumptions; comparisons between the normal, insufficient, and deficient vitamin D groups were evaluated by ANOVAs (with t-tests subsequent to the overall analysis), Wilcoxon rank sum test, χ², or Fisher’s exact test, as appropriate to the data. The relationship of vitamin D levels and psychosis was assessed with an ANOVA type design and the association of psychosis with vitamin D level groups with logistic regression models, assessing race as well as vitamin D level groups in a multivariate model. All analyses were carried out using SAS 9.2 on a Windows 7 platform.

## Results

### Vitamin D deficiency prevalence and association with psychosis

Thirty-five (33.7%) adolescents were vitamin D deficient (<20 ng/ml), and an additional 40 (38.4%) were vitamin D insufficient (20–30 ng/ml). Of those with vitamin D deficiency, 40% had psychotic features compared to only 16% of the sample who were not vitamin D deficient (p < 0.007). Those with D deficiency were 3½ times more likely to have psychotic features (OR 3.52, CI 1.38-8.95, 1df). Of those with normal vitamin D status, 79% (N = 23/29) did not have psychotic features.

### Demographic and other related variable differences

A comparison of demographic variables between adolescents with insufficient, deficient, and normal 25-OH D levels is presented in Table 1.

**Table 1:** Patient Characteristics by 25-OH Vitamin D Levels

	All N = 104	***Deficient (<20 ng/ ml) N = 35***	***Not Deficient N = 69***	p-value*
*Variable*	*X (1SD)* or *n (%)*	*X (1SD)* or *n (%)*	*X (1SD)* or *n (%)*	
**Demographics**				
Age	15.38 ± 1.6	15.28 ± 1.63	15.43 ± 1.60	0.66
Sex (M/F) (%Male)	29/75 (27.9)	11/24 (31.4)	18/51 (27.9)	0.57
Race (White/Black/Hispanic/ Asian/Biracial) (%White)	76/15/1/5/7 (73.1)	17/9/0/5/4 (50.0)	59/6/1/0/3 (67.0)	0.0003
Residence (Urban/ Suburban or Rural) (%Urban)	10/94 (9.6)	3/32 (8.6)	7/62 (6.7)	0.80
Insurance (Private/ Medicaid) (%Private)	65/39 (62.5)	19/16 (54.3)	46/23 (66.7)	0.22
Smoking Status (Yes/No) (%Yes)	18/67 (21.2)	9/20 (31)	9/47 (16.1)	0.11
Inpatient or Outpatient Status (Inpatient/ Outpatient) (% Inpatient)	34/70 (32.7)	14/21 (40)	20/49 (29.0)	0.26
**Metabolic Variables**				
Body Mass Index (BMI)	25.2 ± 7.6	26.1 ± 8.4	24.7 ± 7.2	0.39
Glucose	93.9 ± 18.1	91.7 ± 13.8	94.9 ± 19.7	0.42
Cholesterol, total	167.4 ± 34.4	167.6 ± 30.8	167.3 + 36.7	0.98
High Density Lipoprotein (HDL)	53.1 ± 13.9	54.3 ± 13.5	52.4 ± 14.3	0.63
Low Density Lipoprotein (LDL)	94.0 ± 25.0	95.7 ± 21.9	93.0 ± 26.8	0.71
Triglycerides	96.0 ± 69.3	87.6 ± 57.6	100.5 ± 75.2	0.52
Pulse	79.6 ± 15.6	78.4 ± 12.4	80.3 ± 16.7	0.57
Diastolic Blood Pressure	67.6 ± 9.0	69.1 ± 9.3	66.7 ± 8.7	0.20
Systolic Blood Pressure	115.3 ± 12.7	116.9 ± 14.3	114.5 ± 11.9	0.39
**Illness Features and Family History**				
Psychosis N, (%)	25 (24.0)	14 (40.0)	11 (15.9)	0.007
Immediate Family Psychosis N, (%)	10 (9.6)	4 (11.4)	6 (8.7)	0.66
Extended Family Psychosis N, (%)	10 (9.6)	5 (14.3)	5 (7.3)	0.25
Illness Age of Onset (Prepubertal/ Adolescent) (% Prepubertal)	45/55 (45)	14 (41.2)	31 (47.0)	0.69
**DSM-IV diagnoses, N (%)**				
Bipolar Disorders	37 (36)	13 (37)	24 (34.8)	0.81
Unipolar Depressive	36 (35)	9 (25.7)	27 (39.1)	0.17
Mood Disorder NOS	15 (14)	6 (17.1)	9 (13.0)	0.57
Anxiety Disorder NOS	5 (5)	2 (5.7)	3 (4.3)	0.76
Psychotic Disorder NOS	8 (7.7)	5 (14.3)	3 (4.3)	0.07
Other (Yes/No) (%Yes)	3 (1.3)	0 (0.0)	3 (4.3)	0.21
**Current Medication Exposure, N (%)**				
Stimulants N, (%)	6 (5.8)	2(5.7)	4 (5.8)	0.99
Antidepressants N, (%)	44 (42.3)	11 (31.4)	33 (47.8)	0.11
Antipsychotics N, (%)	35 (33.7)	12 (34.3)	23 (66.7)	0.92
Anticonvulsants N, (%)	18 (17.3)	5 (14.9))	13 (18.8)	0.56
Benzodiazepines N, (%)	6 (5.8)	3 (8.6)	3 (4.35)	0.38
Other medications	31 (29.8)	8 (22.9)	23 (33.3)	0.27
**Past Medication Exposure, N (%)**				
Stimulants N, (%)	18 (17.3)	7 (20.0)	11 (15.9)	0.60
Antidepressants N, (%)	42 (40.4)	12 (34.3)	30 (56.5)	0.37
Antipsychotics N, (%)	21 (20.2)	6 (17.1)	15 (21.7)	0.58
Anticonvulsants N, (%)	20 (19.2)	4 (11.4)	16 (23.2)	0.15
Benzodiazepines N, (%)	3 (2.88)	0 (0.0)	3 (4.35)	0.21
Other medications	9 (8.7)	2 (5.7)	7 (10.1)	0.4

*Evaluated by ANOVA, Wilcoxon rank sum test, χ², or Fisher’s exact test.

Missing data were present for the following (N): BMI, 4;, glucose 5; cholesterol and HDL 50; LDL 49;

TG 49, pulse 4, DBP and SBP 5; calcium 6; height 4; waist circumference 50; weight 3.

#### Racial differences

**Figure 1:**
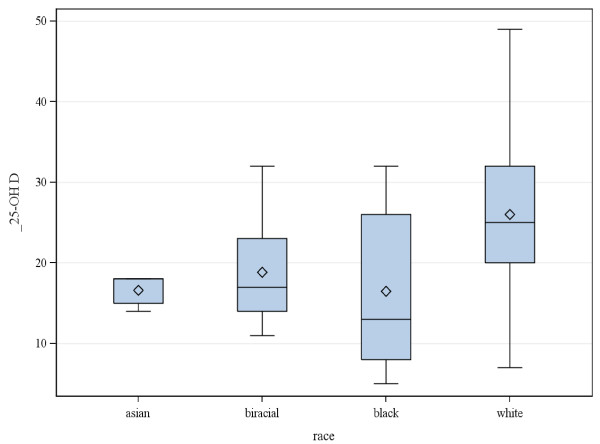
Box-Whisker plot of vitamin D levels by race.

**Figure 2:**
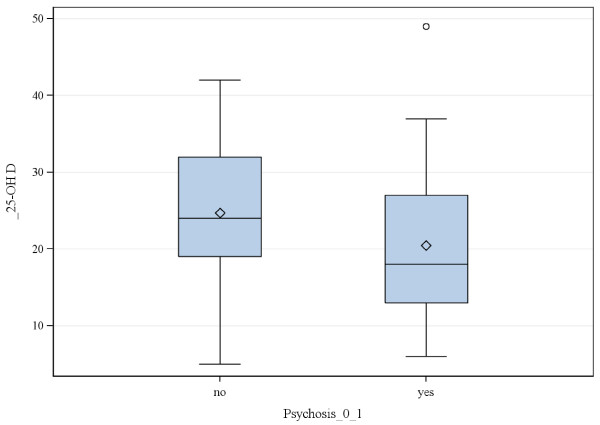
Box-Whisker plot of vitamin D levels by psychosis.

**Figure 3:**
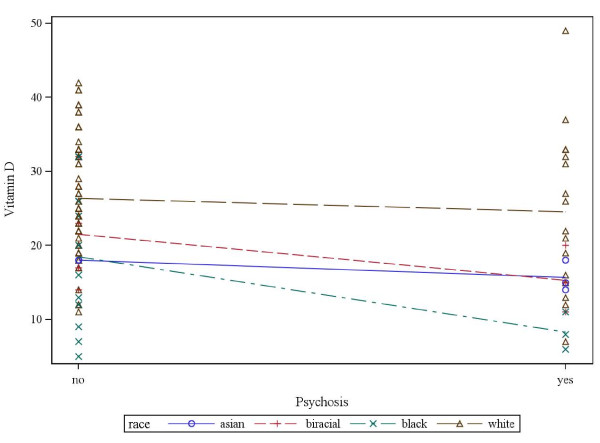
**Association of psychosis, race, and interaction of psychosis and race on vitamin D level expressed as continuous data.** Y-axis displays 25OH-D levels in ng/mL; left vs. right side of figure denotes mean 25OHD levels without and with psychosis by race.

Those who were deficient were more likely to be black or Asian (Figure 1) and have psychotic features (Figure 2). Figure 3 displays the association of 25-OH vitamin D and the interaction of race and psychosis. All groups showed lower 25-OH D levels in the presence of psychosis including Asians who were all deficient. Asian (N = 5) and biracial (N = 7) categories were combined into the “Other” category and Hispanic/Latino ethnicity (N = 1) was combined with Caucasian in the linear modeling that was conducted. Odds ratio comparisons for presence of psychosis with vitamin D deficiency as well as potential covariates, including race and medication exposure, are depicted in Table 2. Psychosis was independently related to race for the “other” group (Asian and biracial individuals) vs. white group, but not for black vs. white groups, nor significantly associated for other vs. blacks. The association of psychosis and vitamin D level was significant overall in the univariate and multivariable analyses. Of added interest was the association of psychosis with Vitamin D levels and race (Table 3). While race and vitamin D levels were associated, race and psychosis were not associated adjusting for vitamin D levels.

**Table 2:** Unadjusted associations between subject characteristics and psychosis

***Variable***	Odds Ratio*	***95% CI***	***P-Value***
Age §	0.91	(0.69, 1.20)	0.51
**25_OH_D §**	**0.95**	**(0.90, 0.99)**	**0.04**
**Vitamin D Deficient vs. Not Deficient**	**3.52**	(1.38,8.95)	**0.008**
BMI §	0.99	(0.94, 1.06)	0.88
CHOL §	0.99	(0.96, 1.01)	0.23
Calcium §	1.70	(0.53, 5.46)	0.38
DBP §	1.01	(0.96, 1.06)	0.73
GLU §	1.02	(0.99, 1.05)	0.07
HDL §	0.98	(0.93, 1.03)	0.40
LDL §	0.99	(0.97, 1.02)	0.71
Pulse §	1.00	(0.97, 1.03)	0.88
SBP §	0.99	(0.96, 1.03)	0.81
Trigl §	0.99	(0.97, 1.01)	0.23
Gender Female vs. Male	0.60	(0.23, 1.58)	0.30
Extended Family Psychosis	2.32	(0.60, 8.99)	0.22
**Immediate Family Psychosis**	**5.92**	**(1.52, 23.11)**	**0.01**
Onset at Adolescence vs. Prepuberty	1.78	(0.68, 4.68)	0.24
Living in Urban vs. Rural/Suburban setting	1.40	(0.33, 5.89)	0.64
Inpatient vs. Outpatient Status	1.22	(0.77, 3.12)	0.69
Private Insurance vs. Medicaid	0.45	(0.18, 1.13)	0.09
Race			0.16
Black vs. White	0.95	(0.24, 3.79)	0.95
Other vs. White **	**3.81**	**(1.08,13.42)**	**0.04**
Smoking (Yes vs. No)	2.92	(0.94, 9.07)	0.06
Current Medication †			
**Antidepressants**	**0.19**	**(0.006, 0.41)**	**0.004**
Antipsychotics	1.44	(0.57, 3.70)	0.44
Anticonvulsants	0.88	(0.26, 2.98)	0.84
Benzodiazepines	0.62	(0.03, 4.08)	0.67
Other Medications	0.89	(0.33, 2.41)	0.82
Past Medication			
Antidepressants	1.22	(0.49,,3.02)	0.67
Antipsychotics	1.81	(0.63, 5.14)	0.27
Anticonvulsants	1.07	(0.35, 3.30)	0.91
Benzodiazepines	6.78	(0.59, 78.15)	0.13
Other Medications	0.89	(0.17,,4.61)	0.89
Stimulants	0.58	(0.15, 2.20)	0.43

Bolded text denotes significance at p < 0.05.

*Odds ratios greater than one indicates increased odds of psychosis.

§ Odds ratio is expressed for a unit increase in the independent variable.

** Other includes Asian and Biracial.

† Current Stimulants evidenced 0 cell count which resulted in questionable model fit.

**Table 3:** Multiple regression model (adjusted) for association of subject characteristics and psychosis

***Variable***	***Odds Ratio***	***95% CI***	***P-Value***
Vitamin D Levels			
Deficient vs Not Deficient	3.26	(1.15 9.19)	0.03
Race			0.33
Black vs. White	0.59	(0.14, 2.6)	0.49
Other vs. White **	1.68	(0.52, 7.67)	0.27
Other vs. Black	3.65	(0.64, 20.83)	0.14

*Odds ratios greater than one indicates increased odds of psychosis.

** White includes Hispanic; Other includes Asian and Biracial.

#### Family history, medication exposure, and seasonal differences

Immediate family history of psychosis and current antidepressant exposure were also independently related to psychosis. Rates of vitamin D insufficiency and deficiency rose from December through March, peaking in March, as would be expected. No seasonal effects, however, were statistically detected (p = 0.14), possibly due to both latitude and high rates of overall deficiency and insufficiency.

## Discussion

This is the first report of an association between vitamin D deficiency in adolescents and severity of mental illness, defined as presence of psychotic features. These findings are similar to a cross-sectional study also linking vitamin D deficiency and adult psychosis [13]. In a study of over 1,000 adults from combined cohorts of a longitudinal evaluation of severe mental illness and a population-based sample from the Oslo Health Study, vitamin D levels and presence of psychosis were compared between native Norwegians and dark-complexion immigrants to Norway. Prevalence of vitamin D deficiency and insufficiency in immigrants with psychosis was 80%, similar to the 72% in our sample of severely mentally ill adolescents. Additionally, 43% of the Oslo community population with psychosis met criteria for vitamin D deficiency, also similar to the 40% of this teen sample with psychosis. In the adult epidemiologic sample, disorientation on the PANSS, weight loss, and lack of physical energy correlated with lower 25-OH D levels after controlling for major depression. Demographics, level of functioning, lifestyle habits, and BMI were not associated with vitamin D levels.

Our findings also agree with an unpublished 2011 report of a child and adolescent psychiatric population residing in the Pacific Northwest. Using the same definitions of deficiency, insufficiency, and normal ranges as in the current study, 21% of 67 youth with severe psychiatric symptoms residing in 2 Oregon residential treatment programs had vitamin D deficiency, vs. 14% of a comparable NHANES sample. For the children with psychotic disorders, the prevalence of vitamin D deficiency was 43% [14]. The overall mean 25-OH D level was 28.9 ng/mL, with 2/3 of the patients falling below the normal range; mean 25-OHD in the youth with psychotic disorders was 26.47 ng/mL (SD12.42). Another group of psychiatric inpatient Parisian adolescents (N = 136) were also found to be largely vitamin D-deficient (72.4%), with the mean 25-OHD value 15–16 ng/mL, lower in blacks and North Africans [15]. No differences in mean levels were found between those taking or not taking antipsychotics, indicating that antipsychotics may not lower vitamin D absorption.

The prevalence of vitamin D deficiency in our sample of acutely mentally ill adolescents is also greater than the high rates observed in U.S. community adolescent populations (34% vs. NHANES 9%)[3] and in Australian adult private psychiatric inpatients (vitamin D deficiency 11%, defined as <25 nmol/L, or 10 ng/mL) [16]. The latter study found a 29% difference between mean levels in patients vs. controls. Our higher prevalence rates of deficiency and insufficiency may be in part due to the latitude of Rochester, NY, 43.145 degrees N. Paris, France is 48.51 N, and Geelong, Australia 38.10 S. Except during summer months, skin makes negligible vitamin D from sunlight at latitudes above 37 degrees north or below 37 degrees south.

### Potential causal mechanisms

Basic and preclinical animal studies provide clues as to how vitamin D may lower risk for psychosis, and how vitamin D deficiency may raise risk for psychosis and depression. These mechanisms include 25-OH vitamin D and calciferol, the renal metabolite of 25-OH D (1,25-OH D): 1) altering neurotrophic factors and monoamine levels [17,18], resulting in vitamin D-related behavioral phenotypes similar to those for depression and psychosis [19], 2) facilitating oxidative stress responses [20], 3) changing multiple neuroendocrine transmitters [21,23], and 4) regulating hormonal and serotonin pathway effects within the CNS [24,25].

### Race, ethnicity, dietary intake and sunlight exposure

Dietary intake and sunlight exposure as sources of vitamin D are influenced by race. The NHANES found poor dietary intake of vitamin D and lower exercise in older African American and female Hispanic adolescents. Although not fully comparable due to different methodologies, our mentally ill non-Caucasian population also demonstrates more vitamin D deficiency, and for Asians and biracial subjects a greater rate of psychosis, but not after adjusting for vitamin D level. Small clinical studies to date suggest potential for a causal link between low vitamin D and mood disorders [8-11,16,26], necessitating that randomized controlled trials in carefully defined populations of interest be performed to better characterize the relationship between vitamin D deficiency and risk for depression and psychosis.

### Alternate hypotheses

In addition to the possibility that vitamin D deficiency contributes to vulnerability to psychosis, other nutritional factors may play a role: adolescents eating a diet low in dairy products may also consume less of other nutrient-rich foods, including those with essential fatty acids. Adverse omega-3:omega-6 fatty acid ratios and/or other dietary micronutrient deficiencies that are commonly associated with vitamin D deficiency such as vitamin B12 may also contribute to emergence of psychotic symptoms.

### Implications for future research in psychiatry

#### Effects on mental illness

If a prospective association between psychotic features and vitamin D deficiency is confirmed, outstanding issues include: 1) how vitamin D affects monoamine function and the HPA axis and immune responses to stress and symptom production, 2) whether supplementation can be protective against incident depression or psychosis and their recurrence, and 3) whether supplementation improves symptoms in those with clinically diagnosed depression or psychosis, especially in populations with darker skin [26]. An open-label Swedish case-series suggests that depression is improved by vitamin D supplementation in adolescents [27]. Prevention studies in high-risk offspring would be highly novel. Thus, future studies could target both prevention of mental and comorbid physical illness, focusing on disparity and somatic treatment augmentation.

#### Importance to overall health in psychiatric populations

Normalizing vitamin D levels warrants study in those with severe mental illness to determine whether, and at what dose, vitamin D helps protect against metabolic side effects from psychopharmacologic treatment or reduces the development of comorbid physical illnesses such as diabetes, cardiovascular disease, and osteoporosis. Low vitamin D is associated with greater BMI, insulin resistance, and systolic blood pressure, lower HDL-C in obese adolescents and adults, and lower final height in young adult women [3,28-31]. Vitamin D status may be especially important in those with serious mental illness as they develop poorer metabolic health at earlier ages. Optimal vitamin D levels also may protect against several different cancers (breast, colon, pancreas, and prostate), and autoimmune disorders (lupus, multiple sclerosis, and Type I diabetes) [32]. Molecular mechanisms of vitamin D that are protective against cancer include reducing cellular oxidative stress [33]. Vitamin D supplementation may thus represent a low-cost population-based intervention capable of reducing utilization of more intensive physical and mental health treatments; multiple trials are underway assessing impact on a variety of physical health conditions including osteoporosis, insulin resistance, and cardiovascular risk. A large scale epidemiologic RCT (the Vitamin D and omega¬3 Trial; VITAL) is examining whether supplementation prevents chronic diseases (http://www.vitalstudy.org).

#### Supplementation issues in adolescents

How should low vitamin D levels be corrected? Supplementation with over-the-counter or prescription vitamin D for those with low vitamin D is important, as natural dietary sources are few and include chiefly oily fish, irradiated mushrooms, egg yolks, and fortified milk, juices, cereal, and margarine (http://dietary¬supplements.info.nih.gov/factsheets/vitamind.asp). Most adolescents, especially those who skip breakfast or are lactose intolerant, do not consume adequate amounts of these foods to maintain optimal vitamin D levels, and cannot meet their requirements through diet alone. Risk for developing vitamin D toxicity with supplements has been largely unsupported [34]; studies giving as much as 14,000 IU per week of D3 to adolescents over one year’s time have shown no evidence of toxicity [35]. A serum level of more than 200 ng/ml 25-OHD may be necessary for symptoms of toxicity to occur. The Institute of Medicine in 2010 raised its daily intake recommendations based on evidence for skeletal growth and maintenance; the current recommended dietary allowance (RDA) is 600 IU per day for 1–70 years of age with an upper level intake of 4,000 IU per day [36]. The American Academy of Pediatrics had previously raised its recommended supplementation from 200 IU in 2003 to 400 IU per day in 2008 to prevent rickets in children and adolescents who do not obtain this goal through fortified foods [37]. Additional initial supplementation to return deficient individuals to normal may be indicated as dietary reference intake amounts recommended by the IOM may still be insufficient for bone health maintenance in many individuals [38]. The amount necessary to prevent breast and colon cancer, Type I diabetes, and multiple sclerosis has been speculated to be closer to 4,000-8,000 IU per day [32]. The amount appropriate for maintenance of best mental and physical health is therefore controversial, and is unknown in the chronically mentally ill, who may metabolize vitamin D more quickly due to added oxidative stress burden.

### Limitations

This work is limited by a small sample size, cross-sectional method, inpatient sampling bias, and lack of formal research measures for diagnosis and severity of illness, family psychiatric history, sun exposure, and intake of Vitamin D and other dietary nutrients. No adolescents, however, were taking vitamin D supplementation. A winter sampling bias is present, potentially contributing to low rates of D-deficiency, however, this does not rule out that seasonal effects in illness severity and admission rates related to greater vitamin D deficiency may occur. Analysis of ultraviolet radiation for latitudes 0 to 80 degrees N found that for March through October, sites from 18 degrees to 44 degrees N (the majority of the continental United States) had equal amounts of vitamin D producing ultraviolet light; November through February only demonstrated decreases in vitamin D producing ultraviolet light [39]. Additional limitations include that adolescents were not screened for osteomalacia; serum parathyroid hormone values were not routinely checked in those who were D-deficient; however, several performed clinically were within normal ranges. Many confidence intervals are wide; confirmation of these results awaits a large sample study and more rigorous design. Probing smaller enriched clinical samples may be useful in providing biologic signals of relevance in populations with depression, for example, using neurocognitive tasks. Descriptive differences in a clinical population compared with population norms may also provide direction for further investigation related to both interactive effects of mental illness and ancestry.

## Conclusion

Vitamin D deficiency is highly prevalent in this descriptive sample of acutely mentally ill adolescents, especially in African-American and Asian teens, and appears related to psychotic symptoms. This work is the first to report an association between psychosis and vitamin D deficiency in psychiatrically hospitalized adolescents [40] and confirms this association found previously in an adult population [13]. Our study also expands on similar deficiency findings from a Parisian inpatient adolescent cohort [15], providing clinical diagnoses of mood disorders in the great majority of inpatient teens with vitamin D deficiency, and further, finding lower risk for psychosis in the presence of antidepressant treatment. Both support the possibility that heightened vulnerability to psychotic features occurs in the substantial proportion of teens with mood disorders who are vitamin D deficient. Prospective trials of vitamin D supplementation are needed to address targeted mental health symptom domains as well as metabolic health variables in D-deficient severely mentally ill adolescents and adults, focusing on dose-finding and tolerability. Calls have been made for clinical monitoring in patients with psychiatric illness as well as randomized trials of vitamin D for depression [41,42]. Clinical screening for vitamin D deficiency in severely mentally ill adolescents is justified by their high risk for both chronic mental illness and early onset of cardiometabolic comorbidities, especially as vitamin D deficiency at mid-life appears a strong independent predictor of all-cause mortality (odds ratios 2.64, 95% CI 1.901 to 3.662. p < 0.0001) [43]. A key clinical question raised by our work and supported by known safety data is whether psychiatrically hospitalized dark-complected adolescents, including African-Americans, Asians, and Muslim females in traditional covered dress, should be routinely supplemented with vitamin D until proven otherwise.

## Competing Interests

The authors declare that they have no competing interests.

## Authors’ Contributions

BLG conceptualized the study, designed the methodology and implementation, interpreted data, and wrote the report. TLF designed the data collection forms, performed the blinded chart review, and assisted in preparing background information. MCF and MP assisted in collecting data. SM provided statistical analysis. All authors read and approved the final manuscript.

## Pre-publication history

The pre-publication history for this paper can be accessed here:

http://www.biomedcentral.com/1471-244X/12/38/prepub
